# Prevalence of mutations associated with tolerance to chlorhexidine and other cationic biocides among Proteus mirabilis clinical isolates

**DOI:** 10.1099/mic.0.001580

**Published:** 2025-07-22

**Authors:** Vicky Bennett, Ocean E. Clarke, Maryam Y. Ravari, James D. Winslow, Matthew E. Wand, Andrew Preston, Emma L. Denham, J. Mark Sutton, Brian V. Jones

**Affiliations:** 1 Department of Life Sciences, University of Bath, Bath, BA2 7AY, UK; 2 Department of Clinical Infection, Microbiology, and Immunology, University of Liverpool, Liverpool, L69 7BE, UK; 3 United Kingdom Health Security Agency, Salisbury, UK

**Keywords:** adaptive evolution, biocides, efflux pumps, lipopolysaccharide, *Proteus mirabilis*, whole-genome sequencing

## Abstract

*Proteus mirabilis* is a frequent cause of catheter-associated urinary tract infection and often exhibits high tolerance to chlorhexidine (CHD), a biocide used widely in healthcare settings. We previously demonstrated that inactivation of the s*mvR* repressor (leading to overexpression of the *smvA* efflux system), truncation of the MltA-interacting protein MipA and aspects of lipopolysaccharide (LPS) structure modulate CHD susceptibility in this organism. However, the prevalence of these mechanisms among *P. mirabilis* clinical isolates, the conditions under which they can be acquired and their impact on susceptibility to other cationic biocides require further study. Through phenotypic and genomic analysis of a panel of 78 *P*. *mirabilis* clinical isolates, we have confirmed that deleterious mutations in *smvR* commonly arise in *P. mirabilis* and are significantly associated with reduced susceptibility to CHD and other cationic biocides. Mutations in *mipA* were also associated with CHD tolerance. Conversely, mutations in *smvA* and the *rppA* response regulator (which governs lipid A modifications that alter LPS surface charge) were associated with increased susceptibility to several biocides. Several isolates harbouring *smvR* mutations displayed incongruous phenotypes, exhibiting relatively modest CHD tolerance, which could not be accounted for by co-occurring mutations in *smvA* and *rppA* or defects in LPS (as assessed by polymyxin B susceptibility). Further analysis of these isolates revealed mutations in the LPS core biosynthesis gene *waaG*, leading to LPS truncation from the inner core region. Directed evolution experiments further reinforced the importance of *smvR* inactivation in biocide adaptation in *P. mirabilis* and demonstrated that relevant mutations can be selected for by exposure to CHD concentrations up to four times lower than the minimum inhibitory concentration. Taken together, these results expand our understanding of mechanisms underlying tolerance to cationic biocides in this species and provide evidence for common mechanisms of cationic biocide tolerance.

## Data Availability

All the raw reads generated through whole-genome sequencing of clinical isolates in this study were deposited in the National Center for Biotechnology Information under Bioproject ID PRJNA1154625. Raw reads from the PR00XXX series clinical isolates are available under Bioproject ID PRJNA475751. The complete annotated genome assembly of the reference strain HI4320 is available under Bioproject ID PRJNA12624. All available clinical metadata and all accession numbers are included in Table S1.

**Impact statement**

The results presented here provide an advanced understanding of mechanisms leading to reduced biocide susceptibility and the evolution of tolerance to antimicrobial agents in clinically relevant pathogens.

## Introduction

Infection prevention and control (IPC) measures are essential to reduce the incidence of healthcare-associated infections and limit the spread of antibiotic resistance in clinical settings [1,2]. Many IPC procedures rely on the use of biocides, such as chlorhexidine (CHD), to control bacterial pathogens in a range of applications [3], including the prophylactic decolonization of various body sites in newly admitted patients [4,5]. In the case of CHD, this important antimicrobial is among the most widely used in healthcare settings and found at concentrations ranging from 0.02% to 4% in products such as wound dressings, lubricating gels, irrigation solutions, catheter maintenance solutions and surgical scrubs [6]. There is also widespread use of CHD in domestic settings, including cleaning products such as surface wipes, hand wash solutions, mouth washes and surface sprays [7].

The increasing reliance of IPC procedures on biocides, coupled with the wide range of in-use concentrations found in various products, leads to important questions regarding the consequences of using these antimicrobials without a clear understanding of how key bacterial pathogens may be affected [8,11]. For example, there is now accumulating evidence that common and problematic bacterial pathogens can adapt to become less susceptible to biocides such as CHD and that this may also promote resistance to some antibiotics and lead to reduced susceptibility to other cationic biocides [12,17]. This raises concerns that inappropriate or overuse of biocides may not only undermine the efficacy of IPC procedures in the future but also select for strains of bacterial pathogens that are more difficult to treat and control [6,8, 18, 19]. However, the specific mechanisms associated with reduced biocide susceptibility in bacterial pathogens and the potential for these to confer tolerance to multiple biocides are not well understood.

The Gram-negative organism *Proteus mirabilis* is a prominent pathogen of the catheterized urinary tract, where it forms unusual crystalline biofilms on catheter surfaces, leading to catheter blockage and the onset of serious clinical complications [20,22]. Since the 1960s, clinical isolates of *P. mirabilis* with relatively low susceptibility to CHD have been described, often with CHD minimum inhibitory concentration (MIC) far exceeding CHD concentrations found in antiseptic products for catheter infection control (CHD MICs ≥512 µg ml^−1^ compared to ~200 µg ml^−1^ in bladder washout solutions) [23]. As a result, these products typically show little efficacy in controlling or preventing catheter-associated *P. mirabilis* infections, and this species is often referred to as CHD ‘tolerant’ or CHD ‘resistant’ [24,26]. However, a wide range of CHD susceptibilities is observed in *P. mirabilis* clinical isolates, indicating that reduced susceptibility to CHD is acquired [16,27]. These features make *P. mirabilis* a useful model organism to study clinically relevant adaptation to biocides.

We have previously demonstrated that clinical isolates of *P. mirabilis* with high CHD MICs (> 512 µg ml^−1^), or isolates adapted to grow at these high CHD concentrations, harbour mutations that inactivate the *smvR* repressor, leading to overexpression of the *smvA* efflux pump [16,27]. Further mutagenesis and directed evolution studies also revealed that aspects of lipopolysaccharide (LPS) and cell envelope structure contribute to CHD tolerance. In particular, disruption of genes involved in LPS inner core biosynthesis increased CHD susceptibility [28]. Adaptation to CHD was associated with the acquisition of mutations in *mipA*, encoding a MltA-interacting cell envelope scaffolding protein, and *rppA*, encoding a response regulator modulating expression of genes responsible for decoration of lipid A with positively charged 4-amino-4-deoxy-l-arabinose (l-Ara4N) moieties [16]. In addition, adaptation of *P. mirabilis* to CHD was also found to modulate susceptibility to octenidine (OCT), suggesting common mechanisms of biocide tolerance [16].

Collectively, these studies indicate that factors inhibiting entry of CHD into the cell work in synergy with upregulation of *smvA* efflux to provide high levels of CHD protection and highlight the potential for acquisition of CHD tolerance to reduce susceptibility to other cationic biocides. However, the prevalence of mutations associated with adaptation to CHD in the wider *P. mirabilis* population and the contribution of these mutations to the modulation of susceptibility to other biocides require further study. To address these questions, we characterized cationic biocide susceptibility in a collection of 78 *P*. *mirabilis* clinical isolates and identified the prevalence of key mutations we have previously linked to CHD tolerance. In doing so, we provide further insight into the mechanisms that underpin reduced CHD susceptibility in *P. mirabilis*, the conditions under which mutations underpinning CHD tolerance can emerge, and the role of these mechanisms in modulating susceptibility to other cationic biocides.

## Methods

### Cell culture and media

Clinical isolates of *P. mirabilis* were obtained from the Royal Sussex County Hospital, Bristol Southmead Hospital, the University of Pittsburgh Medical Center and the UK Health Security Agency (UKHSA) (Table S1). Isolates were cultured in lysogeny broth (LB) broth (10 g l^−1^ tryptone, 5 g l^−1^ yeast extract and 10 g l^−1^ sodium chloride) or tryptic soy broth (TSB) (17 g l^−1^ pancreatic digest of casein, 3 g l^−1^ enzymatic digest of soya bean, 5 g l^−1^ sodium chloride, 2.5 g l^−1^ dipotassium hydrogen phosphate and 2.5 g l^−1^ glucose) with aeration overnight at 37^ °^C. Solid media for the enumeration of single colonies was prepared using LB agar without salt (NSLB, 2% wt/vol). Antimicrobial agents used were chlorhexidine digluconate (CHD) (Sigma-Aldrich), octenidine dihydrochloride (OCT) (Schülke and Mayr), benzalkonium chloride (BZK) (Sigma-Aldrich), cetylpyridinium chloride (CPC) (Fisher Scientific), hexadecylpyridinium chloride monohydrate (HDPCM) (Fisher Scientific) and polymyxin B (PMB) (Fisher Scientific).

### Minimum inhibitory concentrations

MICs for biocides and PMB were determined via broth microdilution in 96-well polypropylene plates (Corning Incorporated) using the methods of Bock *et al*. [29]. Doubling dilutions of each antimicrobial were added to wells of TSB and inoculated with ~10^5^ c.f.u. ml^−1^ of log phase cultures. Plates were incubated statically for 18 h at 37 °C, and then absorbance at OD_600nm_ was measured with a plate reader (Multiskan™ FC Microplate Reader) to determine the MIC, defined as the lowest concentration that prevented detectable growth measured across at least three biological replicates.

### Adaptation of *P. mirabilis* HI4320 to chlorhexidine

The reference strain *P. mirabilis* HI4320 was adapted to CHD through exposure to constant concentrations of CHD. A starting culture of 5 ml LB supplemented with either 8, 16 or 32 µg ml^−1^ CHD was inoculated with 50 µl overnight culture and incubated for 24 h, after which 50 µl was transferred to fresh LB supplemented with the same CHD concentration. This process was repeated every 24 h for a total of eight passages. A non-biocide exposed control (NBC) population was also passaged in the absence of CHD. Cells from each passage and the final adapted populations were frozen in 20% glycerol at −80 °C for storage. This process was completed for three independent replicates for each concentration of CHD exposure. To confirm the stability of the adapted phenotype, cells from each final adapted population were passaged ten times on NSLB agar in the absence of CHD, and the MIC was then tested again to confirm that the adaptation was stable.

### DNA extraction and whole-genome sequencing

Genomic DNA was extracted using the Wizard® Genomic DNA Purification Kit (Promega) as per the manufacturer’s guidance. Purity of each sample was determined using the NanoDrop 2000 (Thermo Scientific), and concentration was determined using the Qubit dsDNA broad-range assay kit and the Qubit 4 Fluorometer (Invitrogen). Samples with a 260:280 ratio of 1.8–2.0 and a 260:230 ratio of 2.0–2.2 were prepared for sequencing. Sequencing was performed using the Illumina NextSeq 2000 platform (Microbial Genomic Services, Pittsburgh), with paired-end read lengths of 150 bp provided as FastQ files for analysis. These were adaptors and quality trimmed using Cutadapt (v4.6) [30] set to a minimum quality of 30. Reads shorter than 45 bp were discarded.

### Identification of genetic variation in clinical isolates and CHD-adapted populations

To identify variation in clinical isolates, Snippy (v4.5) [31] was used to map trimmed reads and call variants against the complete annotated genome sequence of *P. mirabilis* HI4320 [32]. Settings of a minimum base quality of 20, minimum read coverage of 10, minimum allele frequency of 0.9 and minimum mapping quality of 30 were applied. Isolates were assigned to low, medium or high CHD tolerance groups based on MIC_80_ and MIC_20_ values, and the frequency of mutations occurring within each gene of interest within each tolerance group was determined. For the CHD-adapted and NBC populations, polymorphisms were predicted using the breseq pipeline in polymorphism mode [33], and genes containing missense mutations present in the CHD-adapted populations were identified. Mutations that occurred in both the CHD-adapted and NBC populations were disregarded.

### Visualization of LPS core structure

Extraction and visualization of the LPS core were conducted using methods adapted from those of Clarke *et al*. [28]. Overnight cultures were pelleted by centrifugation (2 min, 13,000 ***g***) and washed once in PBS. The washed cell pellet was resuspended in 500 µl of PBS before the addition of 250 µl LPS buffer 1 (0.1875 M Tris pH 6.8, 6% SDS, 30% glycerol). The solution was then boiled for 5 min before being cooled to room temperature. Once cooled, 10 µl of the boiled solution was mixed with 35 µl of LPS buffer 2 (0.0625 M Tris pH 6.8, 0.1% SDS, 10% glycerol, 0.1% bromophenol blue) and 12.5 µl of proteinase K (20 mg ml^−1^, Fisher). The resulting mixture was incubated at 55 °C for 14 h. The prepared LPS lysate was heated to 95 °C for 1 h, and then 5 µl of each sample was mixed with an equal volume of sample buffer (Novex) and loaded onto a 12% Tris-Tricine gel (RunBlue™) and run for 240 mins at 40 V with Tricine-SDS buffer (1M tris, 1M tricine, 1% SDS). The gel was incubated in fixing solution 1 (40% ethanol, 5% acetic acid) overnight at room temperature on an orbital shaker at 60–70 r.p.m. After incubation, fixing solution 1 was replaced with fixing solution 2 (0.7% periodic acid, 40% ethanol and 5% acetic acid), and the gel was incubated at room temperature for 5 min on an orbital shaker at 60–70 r.p.m. The gel was initially rinsed with deionized water and then washed in 1,000 ml of deionized water for 15 min on an orbital shaker at 60–70 r.p.m. This wash step was repeated a further two times before the gel was silver-stained as previously described [34].

### Statistical analysis

Analyses were carried out using GraphPad Prism (v. 10.0.1). To determine the correlation between mutation frequency and biocide tolerance, Spearman rank correlation was used to determine the association between CHD MICs and MICs for other antimicrobials. The Kruskal–Wallis test with Dunn’s correction was used to compare biocide susceptibilities of different isolates. The Kruskal–Wallis test with Dunn’s correction and the chi-squared test for trend were used to identify significant associations between the presence of deleterious mutations in each gene and either a high, intermediate or low tolerance level to each biocide. For all statistical analysis, significance was assigned at *P*<0.05.

## Results

### Biocide susceptibility of *P. mirabilis* clinical isolates

To understand the cationic biocide susceptibility profile in our *P. mirabilis* isolate collection, we first determined the MICs of 78 clinical isolates to CHD, BZK, OCT, CPC and HDPCM (Table 1). As we have previously shown that CHD tolerance in *P. mirabilis* can be influenced by aspects of LPS structure that also modulate susceptibility to PMB [28], the susceptibility of all isolates to this antibiotic was also determined. CHD MICs of these isolates ranged from 8 to >512 µg ml^−1^, congruent with our previous characterization of a small subset of this collection [27]. A similar broad range of MICs was also observed for other biocides evaluated (Table 1). When MIC_50_ and MIC_90_ values were calculated and compared, significant differences between these metrics (significance considered to be ≥4-fold) were observed for CHD, OCT, CPC and HDPCM, which could indicate the presence of sub-populations with notably different susceptibilities for these biocides [35] (Table 1). In contrast, no significant differences between the MIC_50_ and MIC_90_ values were evident for BZK (Table 1). Most isolates were found to exhibit maximal PMB MICs assayed (>2,048 µg ml^−1^), with a minority (20.5%) exhibiting PMB MICs below this value, consistent with the general classification of *P. mirabilis* as intrinsically resistant to this antibiotic (Table 1).

**Table 1.** Cationic biocide susceptibility profile of *P. mirabilis* clinical isolate panel

Antimicrobial*	MIC range (min–max)†	Modal MIC†	MIC_50_†	MIC_90_†	MIC_90_:MIC_50_ Fold difference‡
**CHD**	8 to >512	32 (41.02%)	32	256	**8**
**BZK**	4–128	16 (28.21%)	32	64	2
**OCT**	2–64	2 (62.82%)	2	32	**16**
**CPC**	4 to >512	4 (20.51%)	16	512	**32**
**HDPCM**	4 to >512	4 (24.35%)	32	512	**16**
**PMB**	32 to >2,048	>2,048 (79.49%)	>2,048	>2,048	0

*Cationic biocides tested: CHD, BZK, OCT, CPC and HDPCM. The antibiotic PMB was also evaluated as a general indicator of LPS integrity.

†The MIC range, modal MIC, MIC_50_ and MIC_90_ values for antimicrobials tested are expressed as µg ml−1 concentrations. The MIC range describes the minimum and maximum MIC values observed. For modal MIC values, figures in parentheses show the proportion of isolates with MICs at this value. The MIC_50_ is defined as the concentration at which ≥50% of isolates in a population are inhibited, and the MIC_90_ as the concentration at which≥90% of isolates are inhibited.

‡Fold difference between MIC_50_ and MIC_90_ values. A ≥4-fold difference was considered significant (values in bold).

### Relationship between susceptibility to CHD and other cationic biocides

**Fig. 1.**
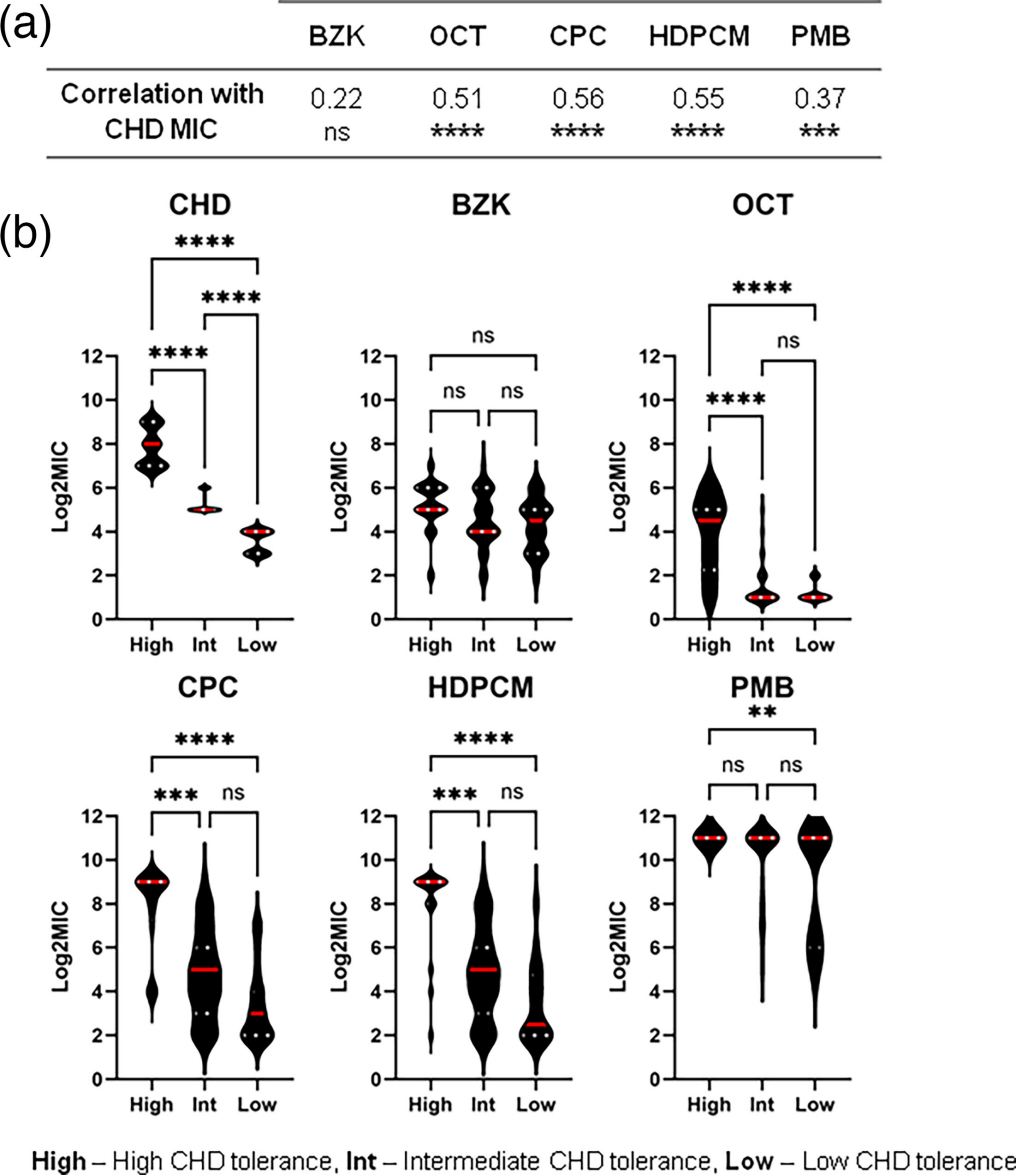
High CHD tolerance is associated with reduced susceptibility to other cationic biocides. (a) Spearman correlation coefficients for CHD Log_2_MICs vs. Log_2_MICs of other biocides and PMB. Coefficients of 0.2–0.39 are indicative of weak correlation, and 0.4–0.59 are indicative of moderate correlation. (**b)** Violin plots showing the overall distribution of Log_2_MICs for isolates categorized based on CHD MIC_80_ and MIC_20_ values. High, high CHD tolerance (≥CHD MIC_80_, ≥128 µg/ml); Int, intermediate CHD tolerance (Int, <CHD MIC_80_ >MIC_20_, <128>16 µg ml^−1^); Low, low CHD tolerance (≤CHD MIC_20_, ≤ 16 µg/ml). Red lines show the median in each group, and white lines show quartiles. MIC_80_ is defined as the concentration at which ≥80% of isolates in a population are inhibited and the MIC_20_ as the concentration at which ≥20% of isolates are inhibited. Significant differences were detected using the Kruskal–Wallis test with Dunn’s correction. ****P≥0.0001; ***P≥0.001; **P≥0.01.

To investigate potential relationships between high CHD tolerance and reduced susceptibility to other biocides and PMB, Log_2_MICs for CHD were correlated with Log_2_MICs of the other antimicrobial agents tested. This analysis revealed moderate but significant positive correlations between CHD susceptibility and susceptibility to OCT, CPC and HDPCM, but no significant correlation between CHD and BZK susceptibility (Fig. 1a). A weak but significant correlation between CHD and PMB susceptibility was also observed (Fig. 1a).

To explore these potential relationships further, we used MIC_80_ and MIC_20_ values for the isolate collection to categorize isolates as high, intermediate or low CHD tolerance and evaluated differences in the distribution of Log_2_MICs between the groups for each antimicrobial (Fig. 1b). Congruent with the basis of this grouping, CHD susceptibility was significantly reduced in intermediate and low CHD tolerance groups (Fig. 1b). The distribution of Log_2_MICs for OCT, CPC and HDPCM in the high CHD tolerance group was also significantly different to intermediate and low CHD tolerance groups (Fig. 1b). In contrast, no significant differences were observed between groups for BZK Log_2_MICs (Fig. 1b). For PMB, a significant difference was observed between the high CHD tolerance group and low CHD tolerance group, in keeping with the previously established impact of LPS defects on CHD susceptibility and the absence of isolates with PMB MICs below >2,048 µg ml^−1^ in the high CHD tolerance group (Fig. 1b). Taken together, these analyses indicate that the majority of isolates with the greatest tolerance to CHD also exhibit significant reductions in susceptibility to OCT, CPC, and HDPCM.

### Association of mutations in *smvAR, mipA* and *rppA* with biocide susceptibility

**Fig. 2.**
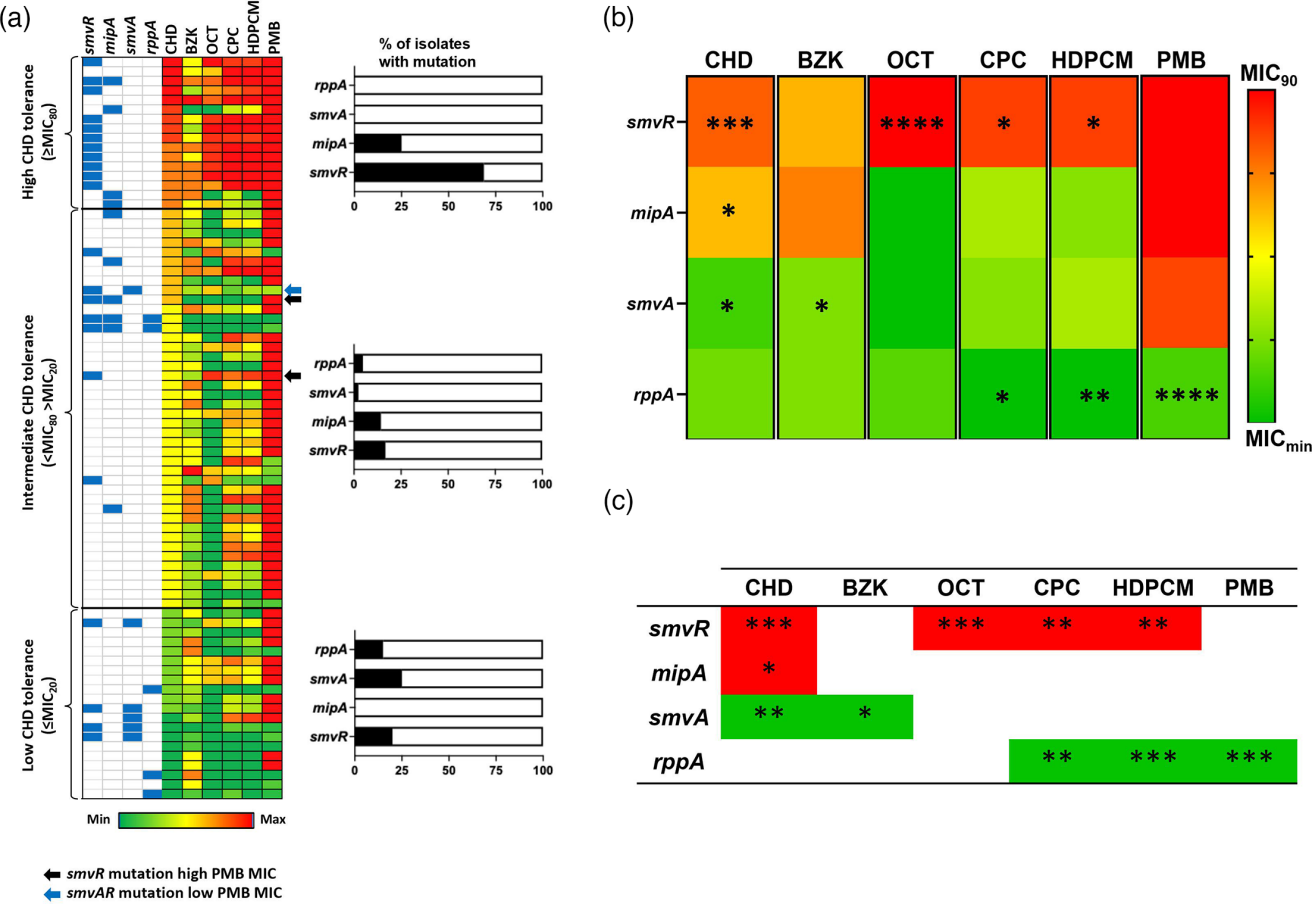
Association of deleterious mutations in *smvAR*, *mipA* and *rppA* with susceptibility to cationic biocides and PMB. The prevalence of mutations in key genes linked with modulation of chlorhexidine susceptibility was calculated for all 78 isolates, and associations with biocide susceptibility were determined. (a) Heatmap showing susceptibility of individual isolates to antimicrobial agents tested when grouped as high (≥CHD MIC80), intermediate (<CHD MIC80 >MIC20) or low (≤CHD MIC20) CHD tolerance. MIC80 is defined as the concentration at which ≥80% of isolates in a population are inhibited and the MIC20 as the concentration at which ≥20% of isolates are inhibited. Associated charts show the proportion of isolates in each group with mutations in each gene. Arrows indicate isolates with CHD susceptibilities inconsistent with identified smvAR mutations and PMB MIC: black arrows, isolates with *smvR* mutations and high PMB MIC expected to exhibit high CHD tolerance; blue arrow, isolate with *smvAR* mutations and low PMB MIC expected to exhibit low CHD tolerance. (b) Heatmap showing median Log2MIC for each biocide in isolates with mutations in *smvR*, *mipA*, *smvA* or *rppA*, respectively. Isolates with co-occurring mutations in both *smvR* and *smvA* were considered as *smvA* mutants only. Heatmap shading reflects median Log2MIC values for isolates with mutations in each gene relative to minimum and MIC90 values of each biocide in the whole population (*n*=78). Significant differences were detected using the Kruskal–Wallis test with Dunn’s correction. (c) Association of mutations with biocide tolerance. Isolates were grouped as high, intermediate or low tolerance for each biocide as for CHD in part (a), and the chi-squared test for trend was used to identify significant associations between the presence of deleterious mutations and biocide tolerance. Significant association with high biocide tolerance is indicated by red shading, while green shading indicates a significant association with low biocide tolerance. ****P≥0.0001; ***P≥0.001; **P≥0.01.

To evaluate the potential for mechanisms involved in CHD tolerance to promote tolerance to additional cationic biocides, we next examined the prevalence of mutations in relevant genes within our isolate collection. Mutations that inactivate the *smvR* repressor (leading to overexpression of the *smvA* efflux system) and *mipA* (encoding the MltA-interacting protein involved in cell wall biosynthesis) have previously been linked to increased CHD tolerance in *P. mirabilis* [27]. Deleterious mutations in *smvA* are, therefore, expected to be associated with increased CHD susceptibility. In addition, mutations predicted to inactivate the *rppA* response regulator (which governs a range of processes including modification of LPS charge through addition of l-Ara4N to lipid A) have been associated with reduced tolerance of *P. mirabilis* to CHD [16,27]. Therefore, the genome sequences of all 78 isolates were analysed for deleterious mutations in the *smvR*, *smvA*, *mipA* and *rppA* coding regions and the predicted promoter sequences for these genes (Fig. 2).

Mutations in *mipA* were significantly associated with reduced CHD susceptibility, with all identified mutations occurring in high and intermediate tolerance groups but absent from low CHD tolerance isolates (Fig. 2a). However, no association was observed between *mipA* mutations and susceptibility to any other biocides tested (Fig. 2b, c). Congruent with the role of the *rppA r*esponse regulator in PMB resistance, mutations in this gene were significantly associated with increased susceptibility to PMB (Fig. 2b, c) and confined to intermediate and low CHD tolerance groups (Fig. 2a). Significant associations with *rppA* mutations and susceptibility to CPC and HDPCM were also evident, but no associations with CHD, OCT or BZK tolerance were identified (Fig. 2b, c).

Most isolates in the high CHD tolerance group (68.75%, 11/16 isolates) were found to have deleterious mutations in *smvR* (Fig. 2a), and the incidence of these was significantly associated with reduced CHD susceptibility (Fig. 2b, c). These included mutations in both the *smvR* CDS as well as mutations within the promoter region. Conversely, 25% of isolates (5/20 isolates) in the low tolerance group had deleterious mutations in *smvA*, and the incidence of these mutations was significantly associated with low CHD tolerance (Fig. 2b, c). Mutations in *smvA* were also absent in high CHD tolerance isolates and rare (2.4% of isolates, 1/42 isolates) within the intermediate CHD tolerance group (Fig. 2a), in keeping with the established role of the *smvAR* system in *P. mirabilis* CHD tolerance [16,27, 28]. However, for other biocides evaluated, the association between *smvAR* mutations and biocide tolerance was less clearly aligned. In the case of OCT, CPC and HDPCM, significant associations between *smvR* mutations and high biocide tolerance were detected, but no corresponding association between *smvA* mutations and increased susceptibility was observed (Fig. 2b, c). For BZK, an association was identified between *smvA* mutations and increased susceptibility; however, no corresponding association was identified between *smvR* mutations and reduced susceptibility (Fig. 2b, c).

Furthermore, *smvR* mutations were not found exclusively in isolates within the high CHD tolerance group but were also present in some isolates assigned to intermediate and low CHD tolerance groups (Fig. 2a). Where low CHD tolerance isolates possessed *smvR* mutations, all also possessed mutations in the cognate *smvA* efflux system which would be expected to negate the impact of *smvR* inactivation on CHD susceptibility (Fig. 2a). In contrast, isolates of intermediate CHD tolerance exhibiting *smvR* mutations did not generally exhibit corresponding *smvA* mutations, but the majority exhibited increased susceptibility to PMB, indicating factors relating to LPS structure are likely to negate the impact of *smvR* mutations in these isolates (Fig. 2a). However, notable exceptions include one isolate with mutations in *smvA* and high PMB susceptibility (indicating defects in both key mechanisms of CHD tolerance assessed here) (Fig. 2a, blue arrow) and two isolates with *smvR* mutations but no corresponding *smvA* mutation or reduction in PMB MIC (Fig. 2a, black arrows). It is also notable that numerous isolates across all groups did not display mutations in any of the genes analysed, indicating that additional uncharacterized mechanisms also commonly modulate biocide susceptibility in *P. mirabilis*.

### LPS truncation increases chlorhexidine susceptibility but does not compromise polymyxin resistance

**Fig. 3.**
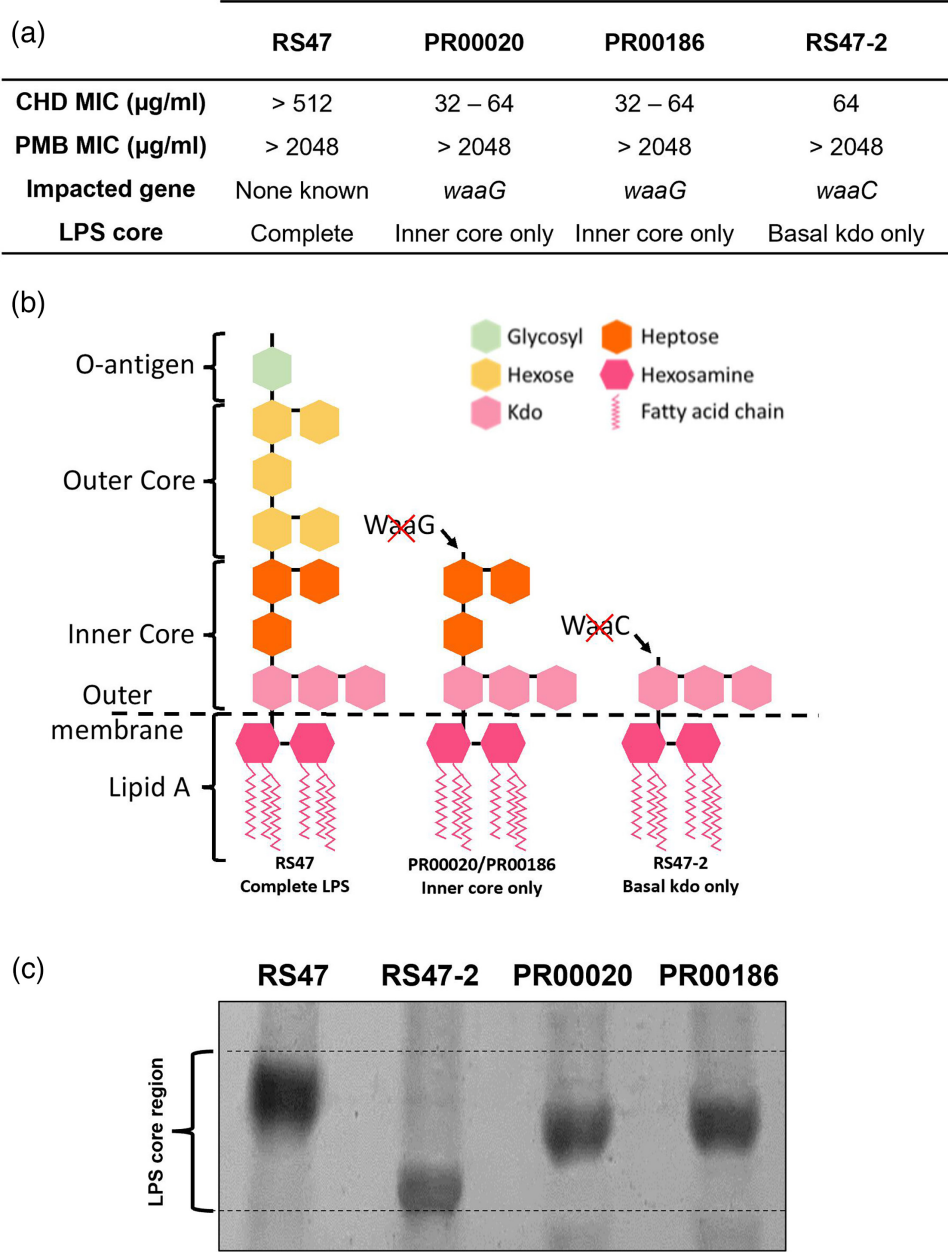
LPS core truncations are associated with reduced CHD susceptibility in isolates with *smvR* mutations and normal PMB susceptibility. The LPS structure of isolates PR00020 and PR00186, which harbour *smvR* mutations and exhibit normal PMB susceptibility (see Fig. 2), was examined and compared to the WT isolate RS47 with high CHD tolerance and the mini-Tn5 derivative RS47-2 with compromised LPS structure and intermediate CHD susceptibility [28]. (**a)** Antimicrobial susceptibility, the presence of mutations in LPS core biosynthesis genes and predicted LPS core structure for strains examined. (**b)** Schematic illustrating predicted effects of *waaG* and *waaC* inactivation on LPS structure. (**c)** Visualization of LPS core structure by Tris-Tricine SDS gel electrophoresis, supporting LPS truncation at outer core region in PR00020 and PR00186.

To identify additional factors modulating biocide susceptibility in isolates where CHD MICs are incongruous with mutations harboured, we undertook further characterization of isolates PR00020 and PR00186 (indicated by positions of black arrows on Fig. 2a). These isolates both harbour *smvR* mutations with no corresponding *smvA* mutations and exhibit low PMB susceptibility. These features are associated with high CHD tolerance in other isolates (CHD MIC 128 to >512 µg ml^−1^) (Fig. 2a), but only moderate CHD tolerance in PR00020 and PR00186 (CHD MICs 32–64 µg ml^−1^) (Fig. 2a, black arrows). Because our previous transposon mutagenesis studies revealed that inactivation of the *waaC* gene leads to LPS truncations that significantly increase CHD susceptibility without altering PMB susceptibility [28], we hypothesized that these isolates may contain analogous naturally occurring mutations that similarly impact LPS structure. To test this, we first surveyed the genomes of these isolates for mutations in genes involved in LPS core biosynthesis. This revealed that both isolates harboured mutations predicted to inactivate the *waaG* gene, a glycosyltransferase which catalyses the linkage of the LPS outer core to the inner core (Fig. 3a, b). To confirm the impact of these mutations on LPS structure, we visualized the LPS core of these isolates and compared this to the highly CHD-tolerant *P. mirabilis* RS47 (complete LPS structure) and its derivative mini-Tn5 *waaC* mutant RS47-2 (fully truncated LPS) (Fig. 3c) [28]. This analysis was consistent with the production of truncated LPS retaining the inner core region in PR00020 and PR00186 (Fig. 3c) and confirms that LPS mutations modulating CHD susceptibility arise naturally in clinical isolates of *P. mirabilis*.

### Sub-MIC exposure can promote high tolerance to CHD and other cationic biocides in *P. mirabilis*

To confirm if adaptation to CHD also leads to tolerance to other biocides tested and better understand the conditions under which relevant mutations can be selected for, we next conducted directed evolution experiments with *P. mirabilis* HI4320. This strain exhibits intermediate tolerance to CHD, with an MIC that corresponds to the MIC_50_ of our wider isolate collection (32 µg ml^−1^) and has a complete and well-annotated genome sequence (NC_010554), making it an ideal reference strain for these studies. In these experiments, HI4320 was passaged in the presence of CHD at MIC and sub-MIC concentrations (8, 16 and 32 µg ml^−1^) to simulate sub-lethal exposure that is likely to occur in real-world conditions. Control populations not exposed to CHD (NBC) were passed alongside these to account for mutations selected by repeated subculture. Using this approach, all adaptation conditions led to significant increases (≥16-fold) in HI4320 CHD MIC, while the MICs for NBC populations were unchanged relative to parental HI4320. For populations exposed to 8 µg ml^−1^ CHD during passage, CHD MICs increased to 512 µg ml^−1^, while those exposed to 16 or 32 µg ml^−1^ exhibited final CHD MICs>512 µg ml^−1^. These phenotypes were stable in the absence of CHD selection.

To identify mutations associated with CHD exposure, genome sequences from adapted populations were compared to the parental genome sequence, and non-synonymous mutations in protein-coding regions or predicted promoter regions were identified. To eliminate mutations that may arise due to the passage of cells only, comparable mutations also identified in the NBC populations were disregarded. This analysis identified mutations in four genes or predicted promoter regions (*smvR*, *mipA*, *fdhF* and *fruK*) present only in CHD-adapted populations (Table 2). Mutations in *fdhF* or *fruK* were not consistently associated with CHD adaptation and occurred at low prevalence in only one population exposed to 8 or 16 µg ml^−1^ CHD (Table S2). In contrast, mutations in *mipA* were identified in the majority of adapted populations exposed to 8 or 16 µg ml^−1^ CHD, and all populations exposed to 32 µg ml^−1^ CHD (Table 2). The majority of mutations in *mipA* were also predicted to inactivate or result in a truncation of this protein.

**Table 2.** Mutations identified in adapted populations

Gene*	P**rotein function**	Mutation location†	Adaptation conditions (µg mL^−1^ CHD)‡		
			8	16	32
*smvR* (PMI_RS14640)	Transcriptional repression of *smvA*	Coding Sequence	Not detected	3/3	3/3
		Promotor	3/3	Not detected	1/3
*mipA* (PMI_RS07300)	MltA-interacting protein involved in cell wall biosynthesis	Coding Sequence	2/3	2/3	3/3
*fruK* (PMI_RS10630)	1-phosphofructokinase	Coding Sequence	1/3	Not detected	Not detected
*fdhF* (PMI_RS14730)	Formate dehydrogenase subunit alpha	Start lost	1/3	Not detected	Not detected

*Gene – genes with mutations identified in CHD-adapted populations under various selective conditions but absent from the associated No Biocide Control (passaged in the absence of CHD exposure). Information in parentheses indicates locus tag in the parental *P. mirabilis* HI4320 genome sequence (NC_010554).

†Mutation location – indicates if mutations in the gene coding sequence or promoter region were identified in adapted populations.

‡Adaptation conditions and mutation prevalence in adapted population – columns indicate the CHD selection for each adaptation, and the number of populations in which mutations were identified. The prevalence and specific positions of mutations in each population are provided in Table S1.

However, the most common and consistent mutations in these adapted populations were in the *smvR* CDS or promoter region, arising in all CHD-adapted populations (Table 2). In populations adapted at the lowest level of CHD selection, only mutations associated with the *smvR* promoter emerged, while mutations in the *smvR* CDS dominated in populations adapted at the higher concentrations of 16 and 32 µg ml^−1^ CHD (Table 2). Notable also in this context was the general increase in the prevalence of *smvR* mutations in populations exposed to higher concentrations of CHD, which corresponds with the key role of *smvA*-mediated efflux in CHD tolerance (Table S1). Taken together, these data further support a key role for the *smvAR* system in CHD tolerance and show that relevant mutations can be selected by low-level CHD exposure. Furthermore, all adapted populations showed significantly increased tolerance to OCT (64-fold), CPC (>16-fold) and HDPCM (>16-fold), but not to BZK, in keeping with the general association between acquisition of CHD tolerance and reduced susceptibility to other biocides observed in the wider isolate panel.

## Discussion

Our analysis of a broad set of clinical isolates shows that deleterious mutations in *smvR* and *mipA* are common among *P. mirabilis* clinical isolates with high tolerance to CHD. Although mutations in these genes have previously been observed in a CHD-tolerant clinical isolate of *P. mirabilis* [27,28], demonstrating that they can arise in real-world settings, these results now confirm that these mechanisms are likely widespread and are present in isolates from both the UK and USA. This work also reinforces the importance of the *smvAR* system in CHD tolerance and the idea that the typical *P. mirabilis* LPS structure (likely in conjunction with the CHD inducible expression of *smvA*) confers a basal level of CHD protection represented by the intermediate CHD tolerance group in this study [27,28]. Our data indicate that this baseline phenotype may be modulated through the acquisition of mutations that either enhance or reduce susceptibility (low and high CHD tolerance isolates, respectively). Mutations upregulating *smvA* expression lead to high-level CHD tolerance, while those compromising *smvA* activity or LPS structure increase susceptibility.

Laboratory experiments with other species have also linked disruptive mutations in *smvR* and its promoter with reduced CHD susceptibility in *Klebsiella pneumoniae* and *oxytoca*, *Salmonella enteritidis*, *Citrobacter freundii *and *Enterobacter cloacae*. Naturally occurring mutations causing truncations in *smvR* have not yet been reported in these species [36,37]. Moreover, typical CHD MICs for *K. pneumoniae* isolates are usually comparable to *P. mirabilis* isolates considered as having low CHD tolerance (8–16 μg ml^−1^) [17,38], indicating that baseline tolerance levels for CHD and other biocides may be higher in the *P. mirabilis* population. This elevated baseline may indicate that *P. mirabilis* is inherently more capable of adapting to pressure from cationic biocides.

*P. mirabilis* isolates exhibiting the greatest CHD tolerance in our panel were also more likely to exhibit reduced susceptibility to other cationic biocides (OCT, CPC and HDPCM), indicating the potential for adaptation to CHD to select for tolerance to multiple biocides. In particular, mutations predicted to inactivate or compromise expression of *smvR* were significantly associated with reduced susceptibility to these biocides, congruent with previous work highlighting the potential contribution of *smvR* inactivation to reduced susceptibility to CHD and other biocides in *P. mirabilis* [16,27].

In this context, the lack of association between *smvA* mutations and tolerance to most biocides was also notable. While this discrepancy may be due to limitations inherent in our isolate collection, where *smvA* mutations were relatively rare, limiting statistical power, this also raises the possibility that *smvR* regulates factors other than *smvA* that are involved in modulating susceptibility to biocides such as OCT, CPC and HDPCM. Alternatively, these data may indicate that the high tolerance to multiple biocides in our high CHD tolerance group is not due to the acquisition of common mechanisms of biocide tolerance, but rather the parallel adaptation of these isolates to tolerate different biocides during distinct exposures in the clinical environment. However, the emergence of elevated MICs for OCT, CPC and HDPCM following adaptation to CHD in our directed evolution experiments argues against this hypothesis and supports the potential for CHD to exposure to select for common mechanisms of biocide tolerance. In particular, the potential role of *smvR* inactivation in acquired tolerance to multiple cationic biocides is supported by both directed evolution experiments and the genomic characterization of isolates.

In contrast to other biocides tested, tolerance to BZK exhibited no significant correlation with CHD tolerance in our isolates, and no association with mutations in *smvR*. Nevertheless, similar mechanisms of BZK tolerance (compared with CHD) have been identified in other Gram-negative species, with efflux upregulation previously linked to BZK tolerance in *Pseudomonas aeruginosa*, *Escherichia coli* and *Salmonella* Typhimurium [13,19, 39], and alterations to membrane surface charge as a result of mutations in lipid A biosynthesis genes also thought to contribute [15,40]. This suggests that the general model for CHD tolerance in *P. mirabilis,* involving the interplay between efflux and LPS structure, is also relevant to BZK tolerance but involves distinct components or patterns of gene regulation. Notably, a significant association between mutations in *smvA* and increased BZK susceptibility was still detected, indicating that normal *smvAR* function and regulation may contribute to modulating the BZK susceptibility in *P. mirabilis*. This concept fits with previous work indicating that repression of *smvA* also increases BZK susceptibility in *P. mirabilis* RS47 [27]. In contrast, studies of *smvA* mutants in other species have shown no changes in BZK susceptibility when either *smvR* or *smvA* is inactivated, and the exact role, if any, of *smvA* in tolerance to BZK requires further study.

The characterization of isolates PR00020 and PR00186 and the identification of naturally occurring LPS truncations modifying CHD susceptibility provide further support for our general hypothesis regarding the role of typical *P. mirabilis* LPS structure in baseline CHD susceptibility and provide new insight into mechanisms modulating susceptibility. Inactivation of *waaG* in these isolates is predicted to inhibit attachment of glucose to the l-glycero-d-manno-heptose-II residue of the inner core, resulting in an inability to synthesize and attach the outer core and O-antigen [41]. In addition, phosphorylation of inner core heptose residues is inhibited in *waaG* mutants, resulting in decreased outer membrane stability due to the reduced electrostatic interaction between divalent cations on the outer membrane and the phosphate groups on the inner core [41,42]. It is also notable that both PR00020 and PR00186 exhibited no increase in susceptibility to PMB, as has been associated with mutants containing similar LPS defects in other species [43,44], indicating this is not a reliable indicator of LPS integrity in *P. mirabilis*.

The phenotypes of these mutants were also consistent with our characterization of a transposon mutant of the highly CHD-tolerant *P. mirabilis* isolate RS47. In this mutant, inactivation of *waaC* resulted in the complete loss of O-antigen and LPS core regions, leading to increased CHD susceptibility [28]. Collectively, these data indicate that defects in LPS structure negate the high-level CHD tolerance otherwise conferred by synergistic effects of the typical *P. mirabilis* LPS structure and efflux upregulation. Although the exact mechanism through which intact *P. mirabilis* LPS may protect against CHD is unclear, it has been hypothesized that entry of CHD into the periplasm requires interaction with the LPS inner core region, which is impeded by O-antigen side chains [45,46]. If so, then loss of O-antigen and outer core in PR00020 and PR00186 would be expected to facilitate interaction between CHD and LPS core regions and increase susceptibility to this biocide [28,45, 46]. However, no clear evidence for a role of LPS truncation in susceptibility to other biocides was provided by these isolates, with each displaying disparate susceptibility profiles to BZK, OCT, CPC, and HDPCM.

Nevertheless, analysis of our isolated panel provided further insight into the role of other LPS modifications in *P. mirabilis* tolerance to CHD and other biocides. We previously linked mutations in the *rppA* response regulator, part of the *rppAB* two-component system, to increased susceptibility to CHD and PMB in the low CHD tolerance isolate RS50a [16]. In *P. mirabilis,* the RppAB system regulates genes in the *arn* operon involved in the biosynthesis and attachment of l-Ara4N to lipid A [47]. The decoration of lipid A with l-Ara4N protects cells from PMB via modulation of net surface charge, which promotes electrostatic repulsion of cationic PMB peptides [47,50]. Disruption of *rppA* or other genes in the *arn* operon has been shown to decrease PMB resistance in *P. mirabilis* and other Gram-negative species [51,53]. It has also been suggested that this mechanism could confer protection from cationic biocides via electrostatic repulsion and has been linked to increased CHD tolerance in *K. pneumoniae* [16,17]. Although this and recent studies indicated no direct role for *rppA* in *P. mirabilis* CHD tolerance, a functional *rppA* appears important for high CHD tolerance [16], and characterization of isolates in this study now indicates a role in modulating susceptibility to CPC and HDPCM.

It is also important to consider the biocide susceptibility of isolates characterized here in the context of real-world biocide use. For example, the NHS routinely uses CHD and OCT in concentrations far higher than MICs reported in this study (0.02% to 4% for CHD, 0.3% for OCT) [54,56], suggesting that biocide susceptibilities observed here would not have clinical relevance. However, real-world use of biocide products for disinfection or antisepsis typically involves application under challenging conditions where exposure time, temperature and organic load substantially influence efficacy [57,58]. Consequently, bacteria are likely to be subjected to sub-lethal biocide exposure, which, as we have demonstrated in our low-level CHD adaptation experiments, can still promote the acquisition of tolerance. Therefore, although the decrease in susceptibility shown by these isolates would theoretically not be enough to protect them against effective treatment at biocide concentrations commonly used, they are likely to provide advantages when real-world scenarios and poor compliance with recommended usage are taken into account. Moreover, the fact that mutations conferring protection against CHD and other biocides have arisen in many of the clinical isolates characterized here supports the hypothesis that the associated reductions in biocide susceptibility are advantageous and underlie the importance of understanding the potential consequences of increased and unchecked biocide use.

## Supplementary material

10.1099/mic.0.001580 Table S1.

## Abbreviations

BZK: benzalkonium chloride
CHD: chlorhexidine digluconate
CPC: cetylpyridinium chloride
HDPCM: hexadecylpyridinium chloride monohydrate
IPC: infection prevention and control
LB: lysogeny broth
LPS: lipopolysaccharide
MIC: minimum inhibitory concentration
NBC: non-biocide exposed control
OCT: octenidine dihydrochloride
PMB: polymyxin B
TSB: tryptic soy broth
